# Seasonal and Age-Associated Pathogen Distribution in Newborn Calves with Diarrhea Admitted to ICU

**DOI:** 10.3390/vetsci8070128

**Published:** 2021-07-09

**Authors:** Engin Berber, Nurettin Çanakoğlu, İbrahim Sözdutmaz, Emrah Simsek, Neslihan Sursal, Gencay Ekinci, Serkan Kökkaya, Ebru Arıkan, Pınar Ambarcıoğlu, Ayşe Gençay Göksu, İhsan Keleş

**Affiliations:** 1 Department of Virology, Faculty of Veterinary Medicine, Erciyes University, Kayseri 38280, Turkey; isozdutmaz@erciyes.edu.tr (İ.S.); serkan.kokkaya@yobu.edu.tr (S.K.); ebruarikan05@gmail.com (E.A.); agencay@erciyes.edu.tr (A.G.G.); 2 Department of Biomedical and Diagnostic Sciences, College of Veterinary Medicine, University of Tennessee, Knoxville, TN 37996, USA; 3 Department of Virology, Milas Faculty of Veterinary Science, Muğla Sıtkı Koçman University, Muğla 48200, Turkey; nurettincanakoglu@mu.edu.tr; 4 Department of Preclinical Science, Faculty of Veterinary Medicine, Erciyes University, Kayseri 38280, Turkey; emrahsimsek@erciyes.edu.tr; 5 Department of Parasitology, Faculty of Veterinary Medicine, Aksaray University, Aksaray 68100, Turkey; neslihansursal@aksaray.edu.tr; 6 Department of Internal Medicine, Faculty of Veterinary Medicine, Erciyes University, Kayseri 38280, Turkey; gekinci@erciyes.edu.tr (G.E.); ihsankeles@erciyes.edu.tr (İ.K.); 7 Department of Microbiology, Faculty of Veterinary Medicine, Bozok University, Yozgat 66700, Turkey; 8 Department of Biostatistics, Faculty of Veterinary Medicine, Hatay Mustafa Kemal University, Hatay 3106, Turkey; kpambarcioglu@mku.edu.tr

**Keywords:** *Cryptosporidium* spp., coronavirus, *E. coli* ETEC K99+, rotavirus, newborn calf diarrhea, neonatal calf diarrhea, intensive care unit, ICU, emergency unit, animal hospital

## Abstract

Calf mortality constitutes a substantial loss for agriculture economy-based countries and is also a significant herd problem in developed countries. However, the occurrence and frequency of responsible gastro-intestinal (GI) pathogens in severe newborn diarrhea is still not well known. We aimed to determine the seasonal and age-associated pathogen distribution of severe diarrhea in newborn calves admitted to the intensive care unit (ICU) of Erciyes University animal hospital over a year. Fecal samples were collected during the ICU admissions, and specimens were subjected to a diarrheal pathogen screening panel that included bovine coronavirus (BCoV), *Cryptosporidium* spp., ETEC K99+, and bovine rotavirus, using RT-PCR and conventional PCR methods. Further isolation experiments were performed with permissive cell cultures and bacterial enrichment methods to identify the clinical importance of infectious pathogen shedding in the ICU. Among the hospitalized calves aged less than 45 days old, the majority of calves originated from small farms (85.9%). The pathogen that most frequently occurred was *Cryptosporidium* spp. (61.5%) followed by rotavirus (56.4%). The frequency of animal admission to ICU and GI pathogen identification was higher during the winter season (44.9%) when compared to other seasons. Most calves included in the study were 1–6 days old (44.9%). Lastly, co-infection with rotavirus and *Cryptosporidium* spp. occurred more frequently than other dual or multi-infection events. This study was the first to define severe diarrhea—causing GI pathogens from ICU admitted newborn calves in Turkey.

## 1. Introduction

Calf mortality is one of the most frequently reported problems related with GI pathogens such as bacteria, viruses, and parasites [1,2]. Moreover, many studies have been conducted to investigate the relation between GI pathogens and newborn calf diarrhea (NCD). Diarrhea has been reported in 19.1% of dairy herds in Dutch farms with the highest frequency (24.0%) in calves aged 1–21 days old [3,4,5]. *Cryptosporidium* spp. (>85% is *C. parvum*), bovine coronaviruses (BCoV), enterotoxigenic *Escherichia coli* K99/F5 (ETEC K99+), and bovine rotavirus are the most frequently identified GI pathogens from calves less than 30 days of age [6,7,8], reported with a prevalence of 17.7–79.9% for coronavirus, 27.8–63.0% for *C. parvum*, 2.6–45.1% for *E. coli,* and 17.7–79.9% for rotavirus [3]. Pathogen-induced diarrhea can occur in calves at different ages depending on the pathogen, but it is most often reported in calves <1 month old [9]. *Cryptosporidium* spp. are most often reported in calves 7–14 days old, coronaviruses in calves 4–30 days old, *E. coli* in calves 1–4 days old, and rotavirus in calves 4–14 days old [10]. It appears that age is the risk factor for infection with different GI pathogens. The season has also been predicted as a risk factor for diarrhea outbreaks [11,12]. Management may also be a reason for scours and mortality. It was later found that the occurrence levels of NCD was most likely associated with birth season [5].

Rotaviruses, classified under the Reoviridae family, are found to be primary causative pathogens for gastroenteritis in both animal and human infections [13,14]. Newborn calves are exposed to rotavirus by the consumption of contaminated milk, water, or feces. Rotavirus can cause damage to enterocytes in the intestine resulting in diarrhea [15]. Rotaviruses also create a potent risk for human health due to its zoonotic potential [15]. Regarding the enteric mucosal cell targeting viral pathogens, BCoV have been commonly identified from newborn calves aged up to 6 weeks, and it is known as a causative viral pathogen of winter dysentery or respiratory complex manifestation among adult cattle herds [16,17,18]. The mode of transmission of BCoV is usually a fecal–oral route, but fecal–nasal or nasal–nasal routes are also possible [19]. The protozoan parasite, *Cryptosporidium* spp., is one of the most common parasites in newly delivered calves [20]. *Cryptosporidium* infection can easily spread in herds by infectious oocysts excreted in feces. Some *Cryptosporidium* species are known to be zoonotic. Humans are usually infected via contaminated food or water sources [21,22,23]. In immunocompromised individuals, infection with *Cryptosporidium* often results in GI-associated symptoms. *Cryptosporidium* can also infect immunocompetent individuals and cause self-limiting diseases. However, the infection can become chronic in immunocompromised individuals [24,25,26,27]. In a study from a farm in Maryland, USA, they observed that among the four *Cryptosporidium* species typically infecting cattle, only *C. parvum* was related to calf diarrhea [28]. In a study by Dubreuil et al. [29], enterotoxigenic *E. coli* (ETEC) was frequently isolated from calves within the first days of life. ETEC bacterial strains produce an adhesion molecule, a fimbriae antigen called K99+, also known as F5, on their surface and attach to small intestine epithelial cells. This leads to an increase in the motility of the lumen by causing the hypersecretion of mucous from the surrounding mucosal epithelial cells in response to secreted toxins from bacteria, thus increasing the severity of the disease [29,30]. Most of the ETEC strains do not secrete these types of toxins but instead secrete K99-expressing ETEC pathotypes, which are identified in neonatal diarrheic calves commonly associated with Sta type heat-labile enterotoxin and considered for neonatal diarrhea [29]. ETEC invades the GI system by the oral route when newborns are exposed to contaminated environments [29].

The aim of this study was to determine the seasonal and age-associated pathogen distribution of severe diarrhea in newborn calves <45 days old admitted to the ICU of Erciyes University animal hospital for a year. We also investigated the co-infection status of calves since co-infections might be affecting the severity of infection.

## 2. Material and Methods

### 2.1. Calves and Sampling

Sample collection was carried out from October 2016 to September 2017 from calves admitted to the emergency unit of the animal hospital at Erciyes University, Turkey. The calves originated from privately owned farms. The size of the farms was noted as small (≤50) or large (≥50). Among the admitted calves, only those <45 days old with severe diarrhea and at least 24 h of ICU hospitalization were included in this study. Regarding the enteric pathogen distribution in age groups, newborn calves were divided into three groups (Figure S1a). Calves were considered diarrheic if feces were abnormally frequent, soft and watery consistency and had bad odor. Clinical scoring and evaluations of disease severity were indexed according to skin test (≥4 s), an inability to stand, sunken eyes, and an inability to eat or drink [31]. ICU admission criteria, including tremors and a rectal temperature below 38.4 °C, were defined by veterinary surgeons specialized in animal internal medicine.

Front-line check-up data and patient information were recorded in a university-owned animal hospital online patient database system (ERUVetO; V.15042019/2015, Kayseri, Turkey) upon registration to the emergency unit of the animal hospital. This system allowed us to retrospectively access further patient information such as date of birth and admission time. Fecal materials were freshly collected from the rectum of diarrheic calves just before starting medication or treatment. Collected samples were stored at −80 °C upon arrival to the diagnostic virology laboratory and were not kept longer than 6 months for genomic material and pathogen isolation studies.

#### Ethics Statement

This study was conducted in accordance with the Declaration of Helsinki, the ARRIVE guidelines, and approved by the committee of the Local Ethics Committee for Animal Experiments office (HADYEK) of Erciyes University and adhered to grant number TOA-2017-7162 with 16/055 release code.

### 2.2. Isolation of Viral RNAs and Molecular Viral Screening from Samples

RNA isolation was performed on stool samples for the investigation of both rotavirus and coronavirus. To perform RNA isolation, a phenol–chloroform protocol was performed. Frozen fecal samples were thawed at room temperature for 30 min and turned upside down several times. Feces were diluted 1:10; w:vol with PBS buffer. After mixing samples by vortex homogenization, they were centrifuged at 10,000× *g* at 4 °C for 10 min. To precipitate the total RNAs including viral genomic strands, RNA isolation was performed as described by Chomczynski et al. [32].

For the identification of rotavirus, one-step RT-PCR was used to target the VP6 region of isolated RNA by mixing 10 µL of RNA with 40 µL of one-step PCR master-mix (TransGen Biotech, Beijing, China). Primer pairs used are listed in Table 1, and previously described by Gómara et al. [33]. The reaction mixture was incubated at 50 °C for 10 min; subsequently, thermal cycle conditions were performed as follows: denaturation at 95 °C for 15 s, annealing at 55 °C for 30 s, and an extension step at 72 °C for 30 s. PCR conditions were completed after 35 cycles of the thermal loop. Initial denaturation at 95 °C for 4 min and a final extension at 72 °C for 7 min were applied before and after heat cycles, and samples were cooled down to 4 °C at the final step. To amplify the bovine coronavirus N region from the RNA samples, one-step RT-PCR was performed on the RNA samples with BCoVF and BCoVR primer pairs (Table 1) and described previously by Cho et al. [34]. Thermal cycle conditions to identify the coronavirus were applied as described above for rotavirus, except the annealing step was performed at 62 °C.

### 2.3. Genomic DNA Extraction from Stool Samples for the Screening of ETEC K99+ and Cryptosporidium *spp.*

Genomic DNA extraction was performed on PBS-diluted stool sample (*n* = 78) supernatants. Briefly, collected fecal specimens were thawed at room temperature (RT) and mixed with PBS (1:10; w:vol). An alkaline phenol DNA extraction method was applied to all samples for DNA isolation for both *E. coli* K99+ (F5) and *Cryptosporidium* spp. screening with minor modifications [35]. Fecal samples were settled at RT for 10 min without centrifugation. To obtain *Cryptosporidium* spp. DNA materials, 320 µL of PBS-diluted fecal liquid was transferred to a centrifuge tube containing 80 µL of lysis buffer (50 mM Tris-HCl, pH 8.5; 1mM EDTA, pH 8; 0.5% SDS). Tubes were subjected to repeated freeze–thaw cycles in chilled ethanol and a 60 °C water bath for 2 min in each cycle. After the disruption of the oocyst wall to release genomic DNA, chloroform/isoamyl alcohol and ethanol precipitation methods were performed as described by Tang et al. [36]. To identify *E. coli* infection, DNA was extracted from feces (*n* = 78) with chloroform/isoamyl alcohol following thawing and dilution in PBS, as previously described by Tang et al. [36]. Air-dried DNA pellets were dissolved in 35 µL of DNase/RNase free water and stored at −20 °C for further analysis. Small subunit (SSU) rRNA gene region-directed nested PCR was applied to DNA samples for the confirmation of *Cryptosporidium* spp. 18SiCF2 and 18SiCR2 primer pairs used for first round amplification (Table 1). The second-round PCR was performed on the first amplified DNA templates along with 18SiCF1 and 18 SiCR1 primer pairs (Table 1). *Cryptosporidium* spp. gene elongation and the DNA amplification protocol were applied as described by Ryan et al. [35]. To detect K99+, a master PCR mix solution targeting the F5 region was prepared as follows: 1XTaq buffer, 0.4 mM MgCl2, 5 mM dNTP, 1 U Taq DNA polymerase (Promega), 1 mM K99F, and 1 mM K99R primers (Table 1) were mixed to a final 20 µL working volume [30].

### 2.4. Isolation of Viral Pathogens in Cell Cultures

MA-104 cell (ATCC, CRL-2378.1) culture was used for rotavirus isolation and virus propagation studies [37,38]. Cells were maintained with 10% fetal bovine sera-FBS (SIGMA, F0926) and 1X antibiotic (Sigma, A5955) containing M199 (Gibco, 11150059) culture growth media conditions. Fecal samples found positive for rotavirus by PCR were diluted 10 times (1:10, vol:vol) with PBS and centrifuged at 10,000× *g* for 10 min at 4 °C. The supernatants were filter-sterilized through 0.22 µm syringe filters (Millipore, SLGVM33RS) to eliminate bacterial and parasitic contaminations. For the activation of rotavirus, trypsin (10 µg/mL) (SIGMA, T1426) was added to filtrates, and samples were pre-incubated at 37 °C on a heat block for 30 min. Trypsin-activated samples were diluted (1:10) in M199 media. A cell monolayer of a MA104 cell line on a 24-well plate was inoculated with trypsin-treated inocula and replicated twice for each. A culture medium was replaced, and virus cultivation in cells was carried out until cytopathic effects (CPE) appeared microscopically up to day 3 of infection. For the isolation of coronavirus from fecal materials, a similar isolation method based on the rotavirus isolation method was applied with minor modifications [39]. MDBK cells were maintained with 10% FBS containing DMEM-F12 (SIGMA, D2906) and FBS concentration was reduced to 1% during the BCoV isolations. The blind virus passages were performed until CPE appeared microscopically. Virus isolations were confirmed by one-step RT-PCR after phenol-chloroform RNA isolation from cell freeze–thawed cells which is described as before by Chomczynski et al. [32].

### 2.5. Isolation of E. coli from Fecal Samples and Colony Screening

*E. coli* isolations were performed on F5-positive samples. Fecal materials were diluted 10-fold in PBS (w:vol; 1:10) and further diluted (Log10) in liquid Luria–Bertani (LB) broth (vol:vol; 1:0) w/o antibiotics (Sigma, Neustadt, Germany). Dilutions were carried out by serially starting from 10^−1^ to 10^−11^. To enrich the bacterial numbers, tubes were incubated on a horizontal shaker at 37 °C for 30 min. The solid LB agar media was prepared without an antibiotic mixture, with the supplementation of sheep whole blood (5%) for colony pick-up isolation [40]. Petri dishes were incubated at 37 °C for 2 days to obtain bacterial colonies. A PCR screening method was performed for *E. coli* K99+ variant according to Francis et al. [40] and Güler et al. [41]. Visible colonies were applied to the colony screening procedure to confirm the isolation of ETEC K99+.

### 2.6. Statistical Analysis 

Graphical illustrations and statistical analysis were performed with Graph Pad Prism 7 software (Graph Pad Software Inc., San Diego, CA, USA). The chi-square (and Fisher’s exact) test was applied to the data to define the p-values of risk factors on the prevalence of each pathogen. Calculations of frequencies and percentages were done in Excel 2016 (Microsoft Excel Software, version 2016, Redmond, WA, USA) through tabular data formulations. A Venn diagram was constructed with online open access software (UGent, Genomics, & 927, 2020).

## 3. Results

### 3.1. Study Population and Inclusion to Study

**Figure 1:**
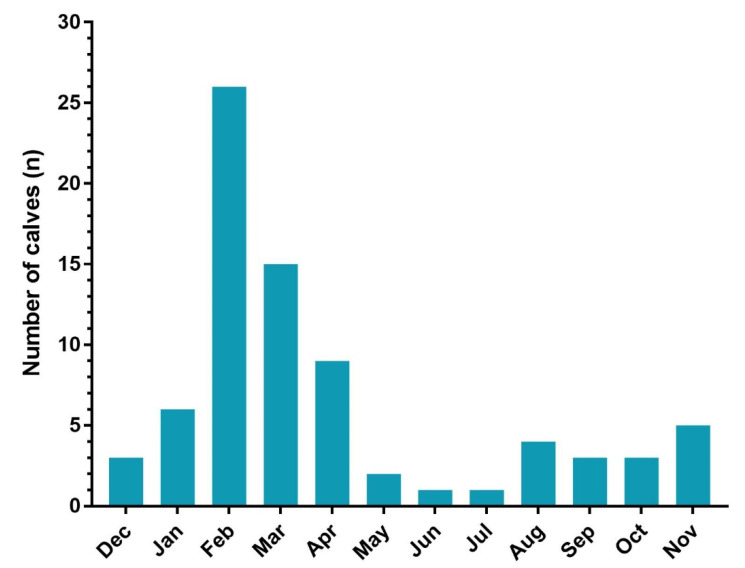
The figure presents the number of calves admitted to ICU and included in the study each month.

During the one-year study period, 78 calves out of 882 calves admitted to the animal hospital met the inclusion criteria and were included in the study. All 78 calves were admitted to the ICU for 24–48 h. Most calves (85.9%) in the ICU originated from small farms and the distribution of bovine species included Simmental (73%), Holstein (15.4%), and Brown Swiss (10.3%), among others (1.3%; cross-breed). Most calves included in the study were males (65.4%). The number of calves admitted each month is shown in Figure 1.

### 3.2. Identified Diarrheal Pathogens 

All calves tested positive by molecular screening for at least one of the four examined pathogens. Molecular identification on isolated RNAs from feces revealed that 56.4% of samples tested positive for bovine rotavirus and 19.2% of calves were positive for coronavirus (Table 2). Molecular identification on isolated DNA from feces revealed that 15.4% of the calves were positive for ETEC K99+ and 61.5% positive for *Cryptosporidium* spp. (Table 2). Molecular screening showed that *Cryptosporidium* spp. was the most identified pathogen followed by rotavirus (Table 2).

### 3.3. Causative Agent Isolation Studies

**Figure 2:**
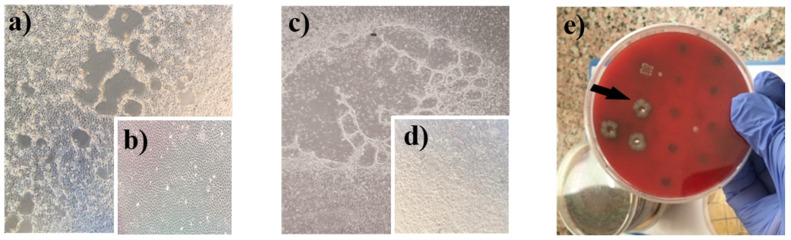
Isolation of infectious diarrheal pathogens from clinical samples. Bovine rotavirus caused the CPE on MA-104 cell monolayers (**a**) and mock-infected cells were intact (**b**). Coronavirus-induced CPE on MDBK cells (**c**) when compared to the mock-infected monolayer (**d**). The black arrow shows the colony picked ETEC K99+ on the solid blood agar dish (**e**).

To understand the infectious stage of diarrhea, we performed the cell culture isolation for viruses and agar isolation method for ETEC K99+. We selected single-infected specimens for this purpose to eliminate the possibility that pathogens could interact and affect each other’s growth and possible isolation. Cell cultures on 15 samples were identified as single-infected with rotavirus by RT-PCR (Table 2) which resulted in 13 rotavirus isolates in MA-104 cells (Figure 2a,b). Virus isolations were confirmed with amplification of the rotavirus VP6 gene by RT-PCR. We also performed MDBK cell cultures for feces from all three BCoV single-infected samples (Table 2), whereof two showed CPE in cell cultures (Figure 2c,d). BCoV isolations were further confirmed with the amplification of the N gene of genomic RNAs by PCR. LB agar cultures on seven fecal samples identified as single-infected with ETEC K99+ by PCR (Table 2) resulted in four ETEC K99+ isolates (Figure 2e). The isolation of ETEC K99+ strains was confirmed by PCR directed to the F5 gene region of K99+ fimbria antigen. The isolation level of viral pathogens in cell culture (coronavirus, 66.7%; rotavirus, 86.7%) and bacterial colony isolation in agar (ETEC K99+ bacterial growth, 57.1%), when compared to PCR findings, indicated that rotavirus; coronavirus; and ETEC K99+ pathogens were still infectious during hospitalization and ICU admission. Isolation studies also indicate that infectious rotavirus shedding is highly excrete (86.7% isolation rate) during acute diarrhea in severe clinical cases while the infectious nature of ETEC K99+ and BCoV reduced in severe cases when calves admitted to ICU.

### 3.4. Risk Factor Analysis and Epidemiological Relevance

**Figure 3:**
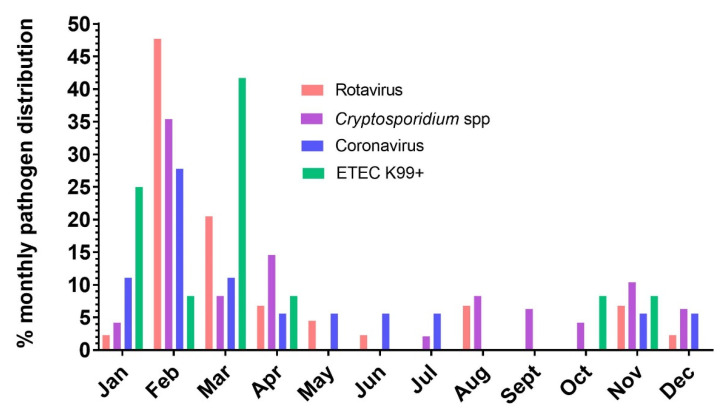
Bar chart: the percentage monthly distribution of pathogens excreted by neonatal calves with diarrhea admitted to the ICU.

The seasonal distributions of calves included in the study are listed in Table 2. Distribution of enteric pathogens in months and seasonal frequencies of pathogens (Figure 3) were also analyzed.

Pathogen distribution analysis revealed that more calves (*n* = 35; age <45 days old) were admitted to the ICU with diarrhea during the winter season compared to other seasons (Table 2). Results indicated that the majority of enteric pathogens were mostly identified during the winter season, from December to February, except for ETEC K99+. Half of the identified ETEC K99+ was found during the spring season, from March to May (50%), and subsequently most of the remaining ETEC K99+ was identified during the winter season (33.3%). The detailed results are assembled in Table 2. Although most calves were admitted to the ICU with diarrhea in the winter months (44.9%), most ETEC positive calves appeared during spring (6 calves/12 positive) (Table 2). Rotavirus, coronavirus, and *Cryptosporidium* spp. occurred in all four seasons. The *Cryptosporidium* seasonal infection rate is significantly different with the highest infection rate in autumn (10/11; 90.9%) (Table S1), followed by summer (5/6; 83.3%). The infection rate did not depend on the season for the other three pathogens (Table S1).

Among the admitted calves with severe diarrhea, most were 1–6 days old (*n* = 35). Severe diarrhea admissions were reduced in the 7–14-day-old group (*n* = 28) and decreased by more than 50% (2.3-fold) in the 15–45-day-old group (*n* = 15). Rotavirus and *Cryptosporidium* spp. distribution in the 1–6-day-old group was estimated close to one another (60 and 54.3%, respectively) and it was measured higher than coronavirus and ETEC K99+ (20 and 31.4%, respectively). No animal in the 15–45-day-old group tested positive for ETEC K99+ (Figure S1a; Table S1), while the ETEC K99+ infection rate was significantly higher (31.4%) in the 1–6-day-old calves than in the 7–14-days-old calves (3.6%). The coronavirus infection rate was significantly higher in calves 15–45-day-old (40.0%) than for calves in the other age groups (Table S1). The single, dual, or multi-infection statuses of calves were evaluated and are presented in Table 2 and in a Venn diagram (Figure S1b) to illustrate the distribution of identified pathogens along with the sample size. Among the examined calves, 55.1% (43/78) were infected with a single pathogen, 37.2% (29/78) were infected with two pathogens, and 7.7% (6/78) were infected with multiple pathogens (Figure S1c). The distribution of pathogens as single, dual, or multi-infection along with the seasonal and age-related distribution of pathogen excretion is summarized in Table S1 along with the p-value estimated by chi-square analysis. The mode of infection of each pathogen reveals that the interaction of pathogens in a single and dual infection mode was more common for all tested enteric pathogens (Figure S1c; Table S1). We did not identify more than three enteric pathogens from the same calve (Figure S1b, Table 2). The most frequent co-infection was the dual infection with *Cryptosporidium* and rotavirus (Table 2). In fact, *Cryptosporidium* appears more often in co-infection (50.0%) than as a single infection (37.5%) (Table S1).

## 4. Discussion 

A major economic cost in agriculture is the loss of newborn calves (USD 1.293 per pre-weaned calf as of 2019 estimation) [42]. The most severe NCDs frequently result in high mortality ratios, even if supportive treatments are administered [3,10]. It has been estimated that calf loss was 15.4% in Turkey due to diarrhea [43]. The frequency and distribution of diarrheal pathogens have been largely investigated and reported in field or farm-collected samples but not in the ICUs of animal hospitals [44,45]. We investigated the occurrence and frequency of common diarrhea-causing pathogens in newborn calves admitted to ICU of Erciyes University from October 2016 to September 2017 in relation to seasonal and age factors. We also investigated whether infections occurred in a single or concurrent manner.

We noticed that 85.9% of calves in the ICU largely originated from small farms. Small farms in the region mostly perform traditional farm activities, and calves are co-housed with their mother or other counterparts in the barns. A recent study in Turkey (2016) showed that more calves (25.2%) from traditional farms had diarrhea than from large farms (10%) [42]. NCD tends to be the biggest problem among small farms in the region of our study (85.9%) when compared to large farms (14.1%). Most diarrheal cases in traditional farming are due to unhygienic practices, poor management, and inadequate treatments. However, we could not evaluate farms for housing conditions, as this study was not conducted at farms. It is also significant that rearing calves individually can decrease diarrheal calves, and such rearing is often practiced by large-scale farm owners (records in the ERUVetO in this study). Another study has shown that bovine rotaviruses and coronaviruses are more often observed in group-housing systems compared with individual housing systems [46]. Individual housing systems are not common in small farms most likely due to their costs.

Our results indicated that males have higher ICU admissions than females (65.4 vs. 34.65%). Species and gender were evaluated as risk factors for newborn diarrhea by Monney et al. [47], and they showed that males are more prone to diarrhea (61.38%) than females (38.62%). Regarding gender differences, Uhde et al. [48] did not find a correlation between genders and calve diarrhea (male, 48.3% vs. females, 51.7%). This is also evidenced in a study by Çitil et al. [49] from the northeast part of Turkey. Çitil et al. [49] also indicated that there was a higher diarrhea incidence in the local bovine breed strain (38%), followed by Simmental (29.9%). We speculated that farmers mostly prefer to breed Simmental cow species for their meat products since the price of meat is higher than that of dairy milk products (based on personal communications with farmers). We did not evaluate either bovine species or sex distribution as a risk factor in this study because there was no homogeneous distribution among them.

The most frequent NCD-causing pathogen in the ICU in our study was *Cryptosporidium* spp. (61.5%). Yildirim et al. [50] investigated the *Cryptosporidium* species distribution in Turkey which also included the same region that the calves originated from in this study. Results showed that 70.8% isolates were *C. parvum*. Moreover, *C. parvum* was the dominant species in pre-weaned calves, especially those with diarrhea [50]. *Cryptosporidium* spp. occurrence was around 20% in a study conducted by Içen et al. [51] in the 2–40-day-old diarrheic calves in the Southeastern Region of Anatolia in Turkey. Uhde et al. [48] estimated that *Cryptosporidium* spp. was the second most frequent (41.7%) diarrhea pathogen in Switzerland, but its statistical importance was equal to that of rotavirus occurrence (55.8%). The reason for the high *Cryptosporidium* spp. (61.5%) occurrence in our study could be related with screened calves’ population, as they originated from ICUs and suffered from severe diarrhea infection. Uhde et al. [48] and some others preferred the use of staining methods or immunochromatographic test kits in their studies. Rotavirus distribution among newborn diarrheic calves was estimated at 55% in Israel [52], 42.7% in Spain [53], and 42% in UK [54]. Altuğ et al. [55] shared the results of an occurrence study on calves aged less than 30 days old in Eastern Anatolia, and they estimated rotavirus occurrence at around 21.56% using lateral flow immunochromatographic test kits. We estimated rotavirus occurrence (56.4%) as the second most frequently identified diarrhea-causing pathogen in calves from our study. The distribution of pathogens can vary among regions. This could be related to differences in herd management and calf rearing practices. Moreover, our study population originated from an ICU with calves critically ill with NCD. BCoV and ETEC K99+ occurrence was 19.2 and 15.4% in this study, respectively. Içen et al. [51] reported BCoV (2.1%) and ETEC K99+ (9.4%) incidences, but results were much lower than in our findings. This could be because of incidences of diarrheal pathogens were more prevalent in severe calves. High occurrence obtained in the present study might also be due to the sensitivity of the diagnostic techniques used.

Brenner et al. [52] reported that the highest occurrence of diarrhea pathogens occurred during winter months. Colder seasons or lower temperatures may be optimal for pathogen survival and infectivity, especially for some viruses. Seasonal factors were retrospectively investigated in pediatric diarrhea in children at the ICU level for up to three years by Chao et al. [56]. The results showed that season was closely related to frequency rates of diarrheal pathogens. They also reported that rotavirus was more prevalent in cold months in the winter season, *Cryptosporidium* spp. often increased after the rainy season, and ETEC strains were higher during the summer season in pediatric hospitalized children [56]. Our results showed that during the winter season, more calves <45 days old (44.9%) were admitted to the ICU with diarrhea compared to other seasons. The outbreak of bacterial diarrhea infections that also involve ETEC, also known as summer diarrhea, was increased in pediatric children in warmer weather [57]. In contrast to children, the ETEC occurrence in newborn calves has been reported at low levels during summer [52]. The reason could be that the management of animals that protects neonates from GI pathogens is more challenging at the herd level in colder seasons compared to warmer weather. Most bacterial infections are seen in human communities in the summer season because of the limited access to fresh and clean water sources. It was reported in Tanzania that inadequate hygienic conditions and storage practices are the source of the enteric pathogens during the summer [58]. However, in the present study, highest admission rates during the winter could be related to calving management systems that farmers are using in the region.

Çitil et al. [49] stated that the highest diarrhea occurrence rates during the winter could be due to the breeding strategies of the farmers. Çitil et al. [49] also speculated that bacterial infections such as *E. coli* occur in newly delivered calves due to exposure to non-hygienic environments immediately after the delivery. This could be related to co-housing the calves with others in small farms. However, further investigations should be addressed to independently identify weather and temperature factors in terms of transmission and persistence around and in livestock communities. However, it remains unknown from this study whether the calving season is a risk factor or not.

The first week of neonatal age was found to be highly significant for newborn diarrheal calves by Demir et al. [42] in the northeast region of Turkey. Similarly, Çitil et al. [49] also reported that 1–7 days old of neonatal age was highly significant and that NCD frequency dropped in the following days. In the present study, the same trend was observed, as most calves included in the study were 1–6 days old (44.9%) and all had diarrhea. Demir et al. [42] reported that enteritis-associated calf death mostly occurred (72.7%) within 1–6 days after delivery. In our study, calves 1–6-day-old group were almost exclusively infected with ETEC. This is consistent with the results reported by Demir et al. [42] who demonstrated that enteritis-associated calf death mostly occurred (72.7%) in 1–6-day-old calves [42]. However, the calves in our study were all alive. As for ETEC, most 1–6-day-old calves (47.7%) were found infected with rotavirus in our study, closely followed by the 7–14-day-old calves (34.1%). This was also observed for rotavirus by Içen et al. [51], as they observed that 66.5% of calves <10 days old became infected. *Cryptosporidium* spp. infection most frequently occurred in the 7–14-day-old (43.8%) calves in our study. However, the infection rate in the 1–6-day-old calves was almost similar (39.6%). Similarly to our findings, Santín et al. [28] reported the highest *Cryptosporidium* spp. prevalence (66.7%) in calves at 2 week of ages.

Severe diarrhea in newborns is often associated with concurrent infections. Rotavirus occurred more often as dual infection with *Cryptosporidium* spp. than other dual infection modes (23.1%) in the present study. The highest single infection status of newborn calves was reported in calves infected with *C. parvum* (28.6%) by Uhde et al. [48] in Switzerland. Içen et al. [51] indicated that the highest single-infected calves were estimated in rotavirus-infected (25%) calves. The occurrence level of the same pathogen in different infection modes (single, dual, or multi-infection) remained unknown. We found that ETEC K99+ primarily occurred as a single infection, however, the incidence of single infection was found to be higher (58.3%) than other infection modes being tested. *Cryptosporidium* spp. and rotavirus occurrence were found higher (23.1%) in dual infection modes, 50% and 52.3%, respectively. The higher distribution of rotaviruses with *Cryptosporidium* spp. with dual infection in the 1–6-day-old (44.4%) and 7–14-day-old (50%) groups (Table 2) is considered an important finding in this study. Uhde et al. [48] reported that dual infection of *C. parvum* with rotavirus reached up to 19.0%, and this was estimated at 15.6% by Içen et al. [51]. Our findings indicate that the occurrence of a single infection of *Cryptosporidium* spp. (23.1%) or dual infection with rotavirus (23.1%) in the ICU was similar. Cruvinel et al. [59] also estimated the occurrence of rotavirus and *Cryptosporidium* in a dual infection mode, and results indicate that rotavirus and *Cryptosporidium* occurrence is correlated to the occurrence levels of each individually, and this correlation is a risk factor for NCD. Furthermore, rotavirus and *Cryptosporidium* infection were positively correlated with coronavirus in a multi-infection mode [59]. In the present study, the occurrence of coronavirus was mostly identified in the dual infection mode (53.3%) and it was estimated to be higher with *Cryptosporidium* spp. (6.4%) than rotavirus (3.8%), similarly to what was reported by Cruvinel et al. [59]. We found that the coronavirus occurrence level was higher than those in the findings of Gumusova et al. [60] (1.96%), Uhde et al. [48] (2%), Içen et al. [51] (1%), and Cruvinel et al. [59] (7.2%). We estimated the total occurrence of coronavirus in the ICU was 19.2% but remained low in the single infection (3.8%) and dual infection cases with rotavirus (3.8%). However, we did not investigate the correlation matrix of these pathogens. In addition, we could not detect coronavirus when neonates become infected with ETEC K99+ and vice versa. Such differences could show that BCoV plays a role in a different stage of NCD. Medina et al. [61] indicated that the viral shedding of rotaviruses decreased 5–6 days after the onset of diarrhea and mortality is more likely associated with the co-infection status of calves. Similarly, our isolation findings indicate that rotavirus is the responsible causative agent in the development of severe acute diarrhea in the ICU, according to the high positivity rate in PCR and isolation success (86.7%) in cell culture. It is also highlighted by Medina et al. [61] that BCoV rapidly replicates in intestinal epithelial cells and causes mass villus loss in the mucosal surface of the lumen more rapidly (40–90 h) after infection. Severe symptoms more likely appear when the virus has disappeared from the GI mucosal surface. The high success of isolation rate (66.7%) and low level of virus positivity in PCR analysis could be related with this rapid and sudden virus replication due to a massive loss of infected villus. This could explain why calves develop severe NCD in the late acute phase of coronavirus infection with a lower positivity rate in both PCR and cell culture isolation studies. The colony isolation rate of ETEC K99+ remained at 57.1% where all isolates were obtained from 1–4-day-old calves. This rapid severe development could be related with antigenic type of ETEC K99+ and number of *E. coli* bacteria ingested. Unfortunately, we did not perform *E. coli* characterization studies. It must be highlighted in this study that rotaviruses and *Cryptosporidium* spp. have a strong correlation that interact in dual infections in newborn neonates aged up to 15 days old (94.4% in a summary of 1–6- and 7–14-day-old calves). These events mainly occurred during the winter season. Farmers engaged in herd management on especially small farms should consider shifting their calving season to a less prevalent season, such as summer and autumn, and proper hygienic practices should be frequently employed while calving occurs. Such practices might include controlling bacterial ETEC transmissions among the animals. Furthermore, few small farms (36%) perform vaccination programs against NCD in the region (ERUVetO records).

## 5. Conclusions

We conclude, due to the high number of admissions to the ICU of an animal hospital during the winter and spring, that the winter season is a risk factor for severe NCD calves. The first week of neonatal age is significant in terms of infection with ETEC K99+. There is also a close relation between rotavirus and *Cryptosporidium* spp. occurrence in a dual infection manner, and this frequently occurred in the first two weeks of neonatal age, especially in the 7–14-day-old group, which is important for *Cryptosporidium* infection. Future direction could include a higher number of calves during several years of investigation to reveal of the importance of acute infections, age, and seasonal impact, which could lead to an understanding of disease prognosis and pathogen correlations.

## Figures and Tables

**Table 1:** The detailed descriptions of primers for calf diarrhea PCR screening panels.

Pathogen	Primer	Target-Loci	Sequence (*5′*-*3′*)	Size (bp)	Ref.
coronavirus	BCoVF	N	GCAATCCAGTAGTAGAGCGT	730	Cho et al. [34]
	BCoVR		CTTAGTGGCATCCTTGCCAA		
*Cryptosporidium* spp.	18SiCF2	SSU rRNA	GACATATCATTCAAGTTTCTGACC	763	Ryan et al. [35]
	18SiCR2		CTGAAGGAGTAAGGAACAACC		
	18SiCF1		CCTATCAGCTTTAGACGGTAGG	587	
	18SiCR1		TCTAAGAATTTCACCTCTGACTG		
*E. coli* K99+	K99F	F5	TATTATCTTAGGTGGTATGG	314	Shams et al. [30]
	K99R		GGTATCCTTTAGCAGCAGTATTTC		
rotavirus	157-R	VP6	GTTTTCCAAGAGTDATHAHYTCAGC	405	Iturriza et al. [33]
	VP6-F		GACGGVGCRACTACATGGT		

**Table 2:** Diarrhea-causing pathogens identified by PCR in feces sample from calves admitted to ICU by age groups, season, and infection status (single, dual, or multi-infections).

Calf Diarrhea Agent and Co-Infections	No. of Positive Calves (%)	Agent Frequency and the Occurrence in Age Groups			Seasonal Distributions of Pathogens			
		1–6 Days Old *n*/Total (%)	7–14 Days Old *n*/Total (%)	15–45 Days Old *n*/Total (%)	Winter *n*/Total (%)	Spring *n*/Total (%)	Summer *n*/Total (%)	Autumn *n*/Total (%)
Total (%)	78 (100)	35 (44.9)	28 (35.9)	15 (19.2)	35 (44.9)	26 (33.3)	6 (7.7)	11 (14.1)
Total infections								
coronavirus (total)	15 (19.2)	7/15 (46.7)	2/15 (13.3)	6/15 (40.0)	8/15 (53.3)	4/15 (26.7)	2/15 (13.3)	1/15 (6.7)
*Cryptosporidium* spp. (total)	48 (61.5)	19/48 (39.6)	21/48 (43.8)	8/48 (16.7)	22/48 (45.8)	11/48 (22.9)	5/48 (10.4)	10/48 (20.8)
ETEC K99+ (total)	12 (15.4)	11/12 (91.7)	1/12 (8.3)	0/12	4/12 (33.3)	6/12 (50.0)	0/12	2/12 (16.7)
rotavirus (total)	44 (56.4)	21/44 (47.7)	15/44 (34.1)	8/44 (18.2)	23/44 (52.3)	14/44 (31.8)	4/44 (9.1)	3/44 (6.8)
Single infection								
coronavirus (only)	3 (3.8)	3/3 (100)	0/3	0/3	1/3 (33.3)	2/3 (66.7)	0/3	0/3
*Cryptosporidium* spp. (only)	18 (23.1)	4/18 (22.2)	10/18 (55.6)	4/18 (22.2)	5/18 (27.8)	7/18 (38.9)	1/18 (5.6)	5/18 (27.8)
ETEC K99+ (only)	7 (9.0)	6/7 (85.7)	1/7(14.3)	0/7	3/7 (42.9)	3/7 (42.9)	0/7	1/7 (14.3)
rotavirus (only)	15 (19.2)	5/15 (33.3)	6/15 (40.0)	4/15 (26.7)	9/15 (60.0)	6/15 (40.0)	0/15	0/15
Dual infection								
coronavirus; *Cryptosporidium* spp.	5 (6.4)	0/5	2/5 (40.0)	3/5 (60)	3/5 (60.0)	0/5	1/5 (20.0)	1/5 (20.0)
coronavirus; ETEC K99+	0 (0.0)	0	0	0	0	0	0	0
coronavirus; rotavirus	3 (3.8)	0/3	0/3	3/3 (100)	0/3	2/3 (66.7)	1/3 (33.3)	0/3
*Cryptosporidium* spp.; ETEC K99+	1 (1.3)	1/1 (100)	0/1	0/1	0/1	0/1	0/1	1/1 (100)
*Cryptosporidium* spp.; rotavirus	18 (23.1)	8/18 (44.4)	9/18 (50.0)	1/18 (5.6)	9/18 (50.0)	3/18 (16.7)	3/18 (16.7)	3/18 (16.7)
ETEC K99+; rotavirus +	2 (2.6)	2/2 (100)	0/2	0/2	0/2	2/2 (100)	0/2	0/2
Multi-infection								
coronavirus; *Cryptosporidium* spp.; ETEC K99+	0 (0.0)	0	0	0	0	0	0	0
coronavirus; *Cryptosporidium* spp.; ETEC K99+; rotavirus	0 (0.0)	0	0	0	0	0	0	0
coronavirus; *Cryptosporidium* spp.; rotavirus	4 (5.1)	4/4 (100)	0/4	0/4	4/4 (100)	0/4	0/4	0/4
coronavirus; ETEC K99+; rotavirus	0 (0.0)	0	0	0	0	0	0	0
*Cryptosporidium* spp.; ETEC K99+; rotavirus	2 (2.6)	2/2 (100)	0/2	0/2	1/2 (50.0)	1/2 (50.0)	0/2	0/2

## Data Availability

No new data were created or analyzed in this study. Data sharing is not applicable to this article.

## Supplementary Materials

The following are available online at https://www.mdpi.com/article/10.3390/vetsci8070128/s1, Figure S1. Representation of age distributions of identified pathogens from respective age groups, Table S1. Chi-square test results for the determination of several risk factors of newborn diarrhea in the ICU.
