# Supplementary material for: Seasonal and Age-Associated Pathogen Distribution in Newborn Calves with Diarrhea Admitted to ICU
Source: Vet Sci. 2021 Jul 9;8(7):128. doi: 10.3390/vetsci8070128 (PMC8310227; doi:10.3390/vetsci8070128)
Supplement: Supplementary file 1 [file vetsci-08-00128-s001.zip › vetsci-1168163-supplementary.pdf]

veterinary sciences

MDPI

Supplementary Materials: Seasonal and Age–Associated Pathogen Distribution in Newborn Calves with Diarrhea Admitted to ICU

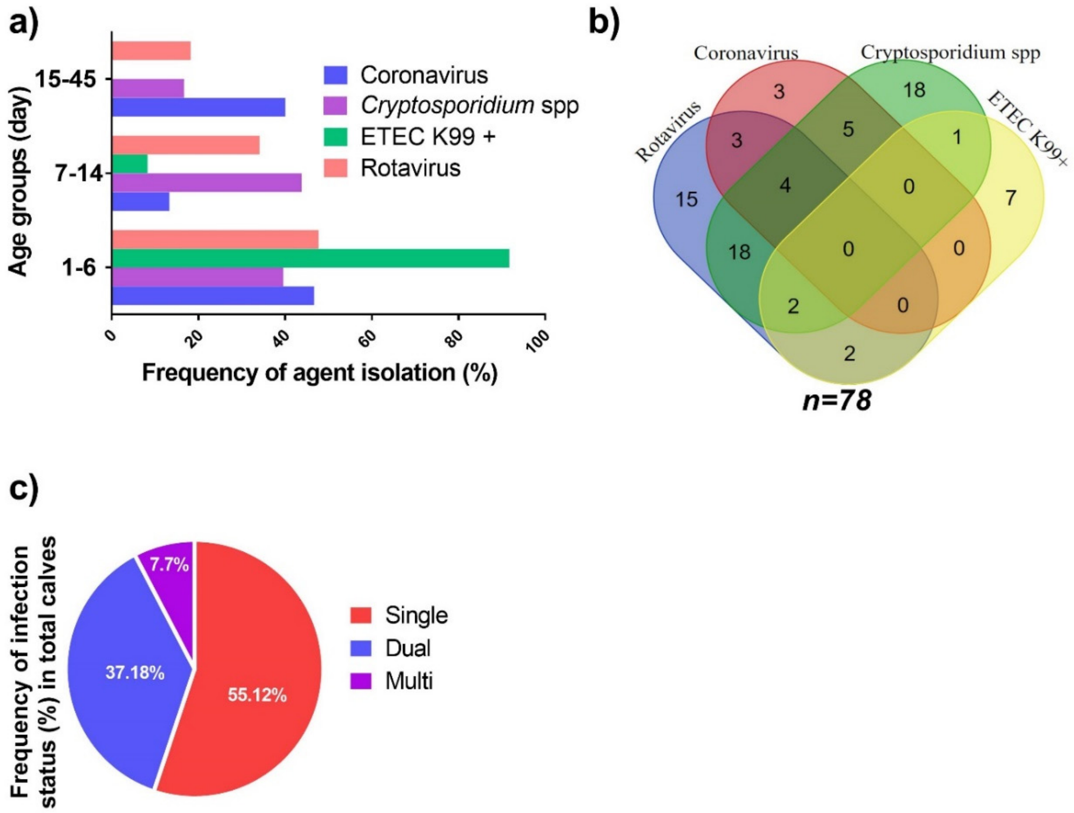

Figure S1. Representation of age distributions of identified pathogens from respective age groups. The respective frequencies for each pathogen (**a**) are shown. A Venn diagram shows the occurrence of pathogens in each calf based on single or co-infection (**b**). A pie chart (**c**) shows the frequencies of mode of infection.

Table S1. Chi-square test results for the determination of several risk factors of newborn diarrhea in the ICU.

Risk Factors		Pathogen Distribution for Each Risk Factor			
		**Rotavirus (*n* = 44)** **_n_/Total (%)**	**Coronavirus (*n* = 15)** **_n_/Total (%)**	**ETEC K99+ (*n* = 12)** **_n_/Total (%)**	***Cryptosporidium* spp.(*n* = 48)** **_n_/Total (%)**
Season	Winter (*n* = 35)	23/35 (65.7)	8/35 (22.9)	4/35 (11.4)	22/35 (62.9)
	Spring (*n* = 26)	14/26 (53.8)	4/26 (15.4)	6/26 (23.1)	11/26 (42.3)
	Summer (*n* = 6)	4/6 (66.7)	2/6 (33.3)	0/6 (0)	5/6 (83.3)
	Autumn (*n* = 11)	3/11 (27.3)	1/11 (9.1)	2/11 (18.2)	10/11 (90.9)
p-value		0.1475 (ns)	0.5641 (ns)	0.4302 (ns)	0.0255 (*)
Age (day)	1–6 (*n* = 35)	21/35 (60.0)	7/35 (20.0)	11/35 (31.4)	19/35 (54.3)
	7–14 (*n* = 28)	15/28 (53.6)	2/28 (7.1)	1/28 (3.6)	21/28 (75.0)
	15–45 (*n* = 15)	8/15 (53.3)	6/15 (40.0)	0/15 (0.0)	8/15 (53.3)
p-value		0.8466 (ns)	0.0332 (*)	0.0018(**)	0.1875 (ns)
Mode of Infection	Single	15/44 (34.1)	3/15 (20.0)	7/12 (58.3)	18/48 (37.5)
	Dual	23/44 (52.3)	8/15 (53.3)	3/12 (25.0)	24/48 (50.0)
	Multi	6/44 (13.6)	4/15 (26.7)	2/12 (16.7)	6/48 (12.5)
p-value		<0.0001 (***)	0.1225 (ns)	0.0724 (ns)	0.0004 (***)
